# Long-Term Outcomes and Prognostic Factors in Metastatic *ALK*-Rearranged Non-Small Cell Lung Cancer Treated with ALK Tyrosine Kinase Inhibitors: Real-World Evidence from a High-Volume Thoracic Diseases Center

**DOI:** 10.3390/cancers18172772

**Published:** 2026-08-26

**Authors:** Abdülkadir Koçanoğlu, Oktay Ünsal, Fatih Kuş, Nesrin Gürçay, Esra Zeynelgil, Yakup Düzköprü, Serdar Karakaya

**Affiliations:** 1 Medical Oncology, Ankara Atatürk Sanatorium Training and Research Hospital, Ankara 06290, Türkiye; droktayunsal@gmail.com (O.Ü.); drserdarkarakaya@gmail.com (S.K.); 2 Department of Pathology, Ankara Atatürk Sanatorium Training and Research Hospital, Ankara 06290, Türkiye; drgurcaynesrin@gmail.com; 3 Medical Oncology, Ankara Etlik City Hospital, Ankara 06170, Türkiye; esra23.05@hotmail.com; 4 Medical Oncology, Aksaray Training and Research Hospital, Aksaray 68200, Türkiye; yakupduzkopru@gmail.com

**Keywords:** non-small cell lung cancer, *ALK* rearrangement, ALK tyrosine kinase inhibitors, prognostic factors, real-world evidence, lorlatinib

## Abstract

**Background/Objectives**: ALK tyrosine kinase inhibitors (TKIs) have improved outcomes in patients with metastatic *ALK*-rearranged non-small cell lung cancer (NSCLC). However, real-world data on long-term outcomes, prognostic factors, and treatment sequencing remain limited. This study aimed to evaluate treatment outcomes, prognostic factors, and lorlatinib efficacy in different treatment settings. **Methods**: We retrospectively analyzed 83 patients with metastatic *ALK*-rearranged NSCLC treated with ALK TKIs at a tertiary oncology center. Survival outcomes, prognostic factors, treatment sequences, and adverse events were analyzed using Kaplan–Meier and Cox regression methods. **Results**: The median follow-up duration was 69.4 months, and the median overall survival (OS) was 73.9 months (95% CI, 58.51–89.32). Median OS was 84.8 months with first-line alectinib and 63.6 months with crizotinib. Median progression-free survival (PFS) durations were 47.5, 23.0, and 14.9 months for alectinib, brigatinib, and crizotinib, respectively. ECOG performance status, histological subtype (adenocarcinoma vs. non-adenocarcinoma), and PD-L1 expression were significant prognostic factors. First-line lorlatinib demonstrated durable disease control, with median PFS not reached and a 24-month PFS rate of 80%. Later-line lorlatinib showed continued activity, with median PFS-2 and PFS-3 of 12.2 and 6.1 months, respectively. Treatment-related adverse events were generally manageable. **Conclusions**: This real-world study provides long-term outcomes and prognostic insights in metastatic *ALK*-rearranged NSCLC. ECOG performance status, histological subtype, and PD-L1 expression were independent prognostic factors for OS. First-line lorlatinib findings suggest durable disease control but remain preliminary and require confirmation with longer follow-up.

## Simple Summary

*ALK*-rearranged non-small cell lung cancer is a specific subtype of lung cancer characterized by changes in the *ALK* gene that can be targeted with ALK inhibitors. Although these treatments have greatly improved patient outcomes, questions remain regarding long-term outcomes and the factors that influence survival in real-world clinical practice. In this study, we evaluated long-term outcomes, prognostic factors, treatment sequences, and safety profiles of ALK inhibitors in patients with metastatic *ALK*-rearranged non-small cell lung cancer treated in a real-world setting. We found that clinical characteristics and tumor features, including performance status, tumor histology, and PD-L1 expression, were associated with patient outcomes. Findings with first-line lorlatinib suggested durable disease control, although these results should be considered preliminary because of the small number of patients and relatively short follow-up. Adverse events related to ALK inhibitors were generally manageable.

## 1. Introduction

Non-small cell lung cancer (NSCLC) accounts for approximately 85% of all lung cancer cases and remains the leading cause of cancer-related mortality worldwide despite significant advances in diagnosis and treatment [1]. Rearrangements of the anaplastic lymphoma kinase (*ALK*) gene are detected in approximately 3–7% of patients with NSCLC and are predominantly observed in younger patients with adenocarcinoma histology and little or no smoking history [2,3]. Although some studies have reported modest differences in the prevalence of *ALK* rearrangements across ethnic populations, overall frequencies appear broadly comparable between Asian and Caucasian patients, with reported variations potentially reflecting differences in clinicopathological characteristics and patient selection [4]. ALK tyrosine kinase inhibitors (TKIs) are targeted therapies that selectively inhibit tumor cells harboring *ALK* rearrangements, while causing minimal damage to normal cells lacking these genetic alterations, thereby exerting potent antitumor activity [5]. The introduction of ALK TKIs into clinical practice has fundamentally changed the treatment approach for this patient population, replacing cytotoxic chemotherapy as the preferred treatment strategy.

Crizotinib, the first-generation ALK TKI, was the first targeted agent to demonstrate clinical benefit and was subsequently incorporated into the treatment of patients with metastatic NSCLC harboring *ALK* rearrangements [2]. Subsequently, the second-generation ALK TKI alectinib became widely adopted after demonstrating superior progression-free survival (PFS) and overall survival (OS) outcomes compared with crizotinib, together with substantial intracranial activity in patients with brain metastases [6,7]. Brigatinib and ceritinib are also second-generation ALK TKIs with demonstrated efficacy, including in patients who developed resistance after crizotinib treatment [8,9]. Brigatinib has shown superior PFS and OS compared with crizotinib and has also demonstrated improved intracranial activity in patients with brain metastases [10].

The introduction of lorlatinib, a third-generation ALK TKI, following the results of the CROWN trial has significantly influenced treatment strategies for patients with metastatic *ALK*-rearranged NSCLC and has generated ongoing discussion regarding optimal treatment sequencing. In this trial comparing lorlatinib with crizotinib, the median PFS was 9.1 months in the crizotinib arm, whereas the lorlatinib arm had not reached median PFS at the 7-year follow-up. These remarkable results have positioned lorlatinib as one of the most effective ALK TKIs for first-line treatment [11,12]. Furthermore, lorlatinib has demonstrated clinically meaningful activity in patients previously treated with second-generation ALK TKIs, particularly against resistance mutations emerging after second-generation ALK TKI therapy [13].

In most clinical trials evaluating ALK TKIs, crizotinib has served as the comparator arm. However, prospective studies directly comparing second- and third-generation ALK TKIs are limited. This uncertainty raises important clinical questions regarding treatment selection and the optimal sequencing strategy for ALK TKIs. Furthermore, real-world evidence is needed to evaluate treatment outcomes in patient populations that are often underrepresented in clinical trials and to identify prognostic factors that may influence survival and guide treatment decisions. In this study, we retrospectively evaluated real-world outcomes of patients with metastatic *ALK*-rearranged NSCLC treated at a high-volume thoracic diseases center in Türkiye. We aimed to assess OS and PFS outcomes, identify prognostic factors associated with survival, and evaluate the safety profile of ALK TKIs. Additionally, we investigated the real-world effectiveness of lorlatinib in later treatment lines among patients who had previously received first-line therapy with first- or second-generation ALK TKIs.

## 2. Materials and Methods

### 2.1. Data Collection and Patient Follow-Up

A retrospective analysis was conducted including patients with metastatic NSCLC harboring *ALK* rearrangements who were followed and treated at the Medical Oncology outpatient clinics of Ankara Atatürk Sanatorium Training and Research Hospital and initiated first-line ALK TKI therapy between 1 January 2016 and 31 January 2026. The selection of ALK TKI during the study period was based on treating physician preference and was influenced by the timing of the introduction and availability of individual ALK TKIs in Türkiye. No additional molecular profiling, pharmacogenomic analysis, or drug-sensitivity testing beyond confirmation of *ALK* rearrangement was used to guide the selection of a specific ALK TKI. Patients were eligible if they were aged ≥18 years, had a pathologically confirmed diagnosis of NSCLC, had documented *ALK* rearrangement, received an ALK TKI as first-line treatment for metastatic disease, and had complete follow-up data available in medical records and electronic databases. Patients with a history of a second primary malignancy were excluded. A total of 95 patients diagnosed with *ALK*-rearranged metastatic NSCLC were initially screened. Of these, 12 patients were excluded: five because they received chemotherapy rather than an ALK TKI as first-line treatment, two because of a history of a second primary malignancy, and five because of incomplete clinical or follow-up data. Accordingly, 83 patients were included in the final analysis.

Clinical and demographic characteristics, including age, sex, smoking history, Eastern Cooperative Oncology Group performance status (ECOG PS), pathological subtype, predominant adenocarcinoma subtype, stage at diagnosis, programmed death-ligand 1 (PD-L1) expression status, comorbidities, metastatic sites, ALK TKIs and other systemic treatments received in different treatment lines, treatment initiation dates, dates of disease progression, last follow-up dates, and dates of death were collected from medical records and electronic databases. For patients with metachronous metastatic disease, information regarding prior curative-intent treatments, including surgery, chemotherapy, and chemoradiotherapy, was also collected.

*ALK* rearrangement was considered positive when detected by at least one of the following methods: immunohistochemistry (IHC), fluorescence in situ hybridization (FISH), or next-generation sequencing (NGS). Disease progression was primarily determined based on radiology reports and the treating clinician’s assessment documented in routine clinical practice. Radiological assessments were generally performed at intervals of approximately 10–14 weeks, although the timing varied because of the retrospective nature of the study. In cases where progression status was uncertain, available radiological images were retrospectively reviewed according to RECIST version 1.1. However, complete imaging data were not available for all patients, precluding uniform retrospective RECIST-based reassessment of the entire cohort. PFS was defined as the time from initiation of first-line ALK TKI therapy to documented disease progression or death from any cause, whichever occurred first. For subsequent-line ALK TKI treatment, PFS-2 and PFS-3 were defined as the time from initiation of the second- and third-line ALK TKI, respectively, to documented disease progression or death from any cause, whichever occurred first. Patients without progression or death were censored at the date of their last disease assessment. OS was defined as the time from initiation of ALK TKI therapy to death or the last follow-up date.

For the safety analysis, all patients who received the study drug at any line of metastatic treatment (first-line, second-line, or later-line) were included, regardless of the treatment sequence. Adverse events were evaluated across the entire cohort of patients exposed to the drug.

The study protocol was approved by the Scientific Research Ethics Committee of Ankara Atatürk Sanatorium Training and Research Hospital, Ministry of Health (approval number: 2024-BÇEK/320; date: 9 July 2025). The study was conducted in accordance with the principles of the Declaration of Helsinki.

### 2.2. Statistical Analysis

Statistical analyses were performed using IBM SPSS Statistics version 22.0 (IBM Corp., Armonk, NY, USA). The clinical and demographic characteristics of the patients were analyzed using descriptive statistics. Categorical variables were presented as numbers and percentages (n, %), whereas continuous variables were expressed as mean ± standard deviation if they showed a normal distribution, and as median and range (minimum–maximum) if they did not show a normal distribution.

For survival analyses, the Kaplan–Meier method and the log-rank test were used for univariate analysis, whereas the Cox proportional hazards regression model was used for multivariate analysis. PD-L1 expression was categorized as <1%, 1–49%, and ≥50%. Patients with unavailable PD-L1 data were excluded from univariable and multivariable analyses involving PD-L1, and no imputation was performed for missing values. The reverse Kaplan–Meier method was used to calculate the median follow-up time. Parameters found to be significant at a *p*-value < 0.05 in the univariate analysis were included in the multivariate analysis. A *p*-value < 0.05 was considered statistically significant for all statistical analyses.

## 3. Results

### 3.1. Patient Characteristics

A total of 83 patients were included in the study, including 45 (54.2%) males. The median age at diagnosis was 55 years (range, 26–77). Thirty-one patients (37.3%) had at least one comorbidity. The number of patients with adenocarcinoma histology was 74 (89.2%). Among patients with adenocarcinoma, the predominant subtype was known in only 24 patients (28.9%). These consisted of solid predominant subtype in 9 patients, acinar predominant subtype in 8 patients, micropapillary predominant subtype in 3 patients, papillary predominant subtype in 2 patients, and signet ring cell subtype in 2 patients. Mucin staining was positive in 32 patients (38.6%) and negative in 30 patients (36.1%). The number of patients with de novo metastatic disease was 65 (78.3%). The most common metastatic site was pleural metastasis, observed in 50 patients (60.2%), followed by distant lymph node metastasis in 33 patients (39.8%) and bone metastasis in 33 patients (39.8%). Brain metastases were present in 23 patients (27.7%). Contralateral lung metastasis and liver metastasis were observed in 15 (18.1%) and 13 (15.7%) patients, respectively. Among the 18 patients with metachronous metastatic disease, 10 (55.6%) had previously undergone surgery with curative intent, of whom seven received adjuvant chemotherapy. The remaining eight patients (44.4%) had received definitive chemoradiotherapy before the development of metastatic disease. ALK rearrangement was identified using FISH in 70 patients (84.3%), IHC in 12 (14.5%), and NGS in nine (10.8%). Because more than one testing method was used in some patients, these categories were not mutually exclusive. Notably, *ALK* rearrangement was confirmed by FISH in all nine patients with non-adenocarcinoma histology; three of these patients also showed *ALK* positivity by IHC. Alectinib was the most frequently used agent in first-line treatment, administered to 42 patients (50.6%). The number of patients receiving crizotinib, lorlatinib, and brigatinib as first-line treatment was 19 (22.9%), 15 (18.1%), and 7 (8.4%), respectively (Table 1).

### 3.2. Follow-Up

The median follow-up duration estimated by the reverse Kaplan–Meier method was 69.4 months (95% CI, 56.28–82.62) in the overall cohort. In subgroup analyses, the median follow-up duration was 74.1 months (95% CI, 68.17–80.19) in patients receiving alectinib, 114.8 months (95% CI, 69.30–160.47) in patients receiving crizotinib, 18 months (95% CI, 16.78–19.21) in patients receiving lorlatinib, and 43 months (95% CI, 7.74–78.33) in patients receiving brigatinib.

### 3.3. Overall Survival

**Figure 1:**
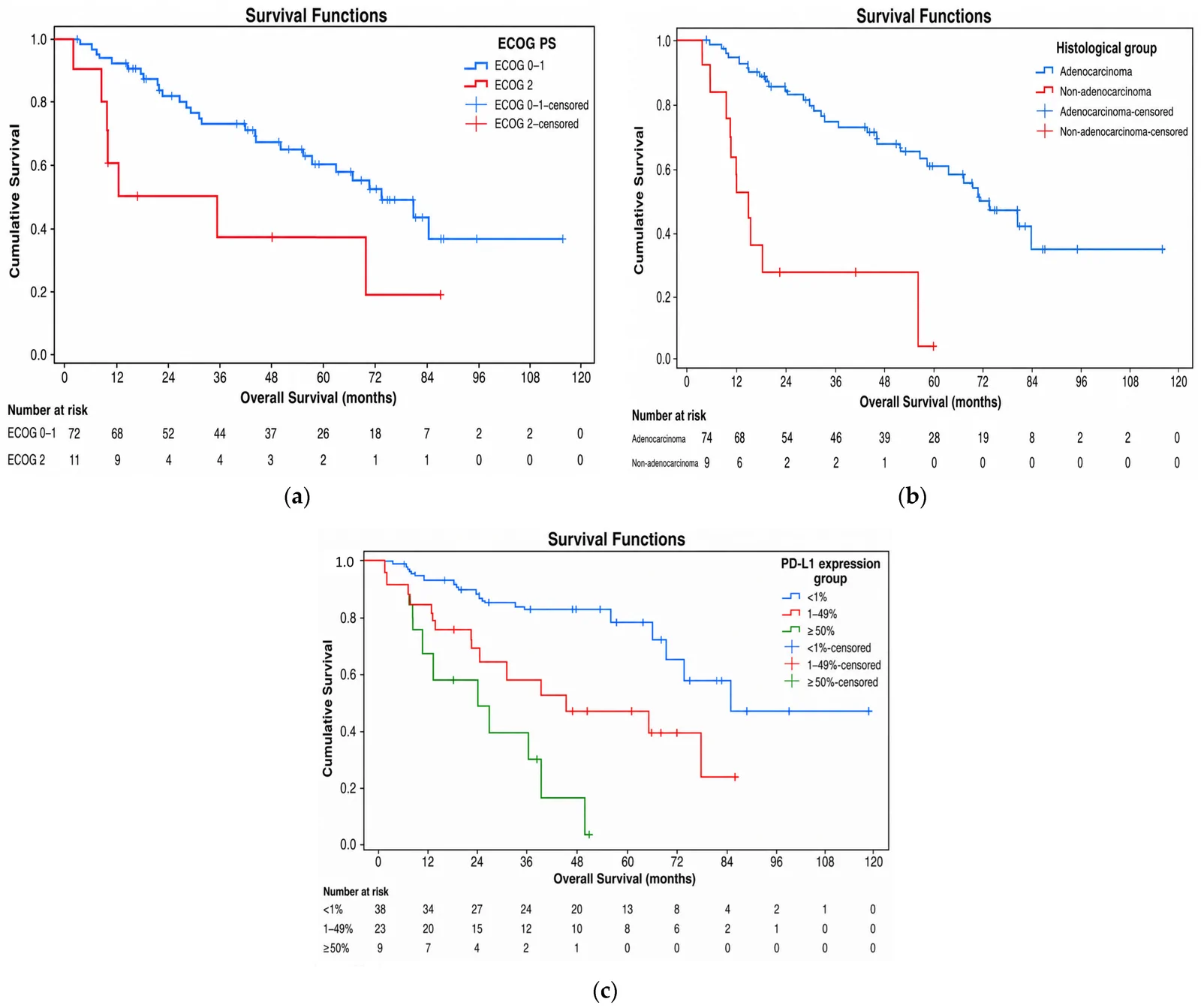
Kaplan–Meier curves for overall survival according to prognostic factors: (**a**) ECOG performance status; (**b**) histological group; and (**c**) PD-L1 expression status.

In the overall cohort, the median OS was 73.9 months (95% CI, 58.51–89.32). In patients receiving first-line lorlatinib for metastatic disease, the median OS was not reached (NR) at the time of analysis. The median OS was 36.8 months (95% CI, 11.17–62.48) in patients receiving brigatinib and 63.6 months (95% CI, 47.58–79.55) in patients receiving crizotinib. The median OS in the alectinib group was 84.8 months (95% CI, NR–NR). At 24 months, 86.6% of patients receiving lorlatinib remained alive. Only the comparison between the alectinib and brigatinib groups showed a nominally significant difference (*p* = 0.037). At the time of data cutoff, 35 of 83 patients (42.2%) had died. The numbers of deaths were 16 of 42 patients (38.1%) in the alectinib group, 13 of 19 (68.4%) in the crizotinib group, four of seven (57.1%) in the brigatinib group, and two of 15 (13.3%) in the lorlatinib group. In univariable analyses for OS, statistically significant differences were observed according to ECOG performance status (*p* = 0.020), histological subtype (adenocarcinoma vs. non-adenocarcinoma) (*p* = 0.001), PD-L1 expression status (*p* = 0.004), and brain metastasis status (*p* = 0.035). In multivariable analysis, brain metastasis lost statistical significance (*p* = 0.182), whereas ECOG performance status (*p* = 0.001), histological subtype (adenocarcinoma vs. non-adenocarcinoma) (*p* = 0.002), and PD-L1 expression status (*p* < 0.001) remained statistically significant (Table 2). Kaplan–Meier curves of variables with prognostic significance for OS are presented in Figure 1.

### 3.4. Progression-Free Survival

**Figure 2:**
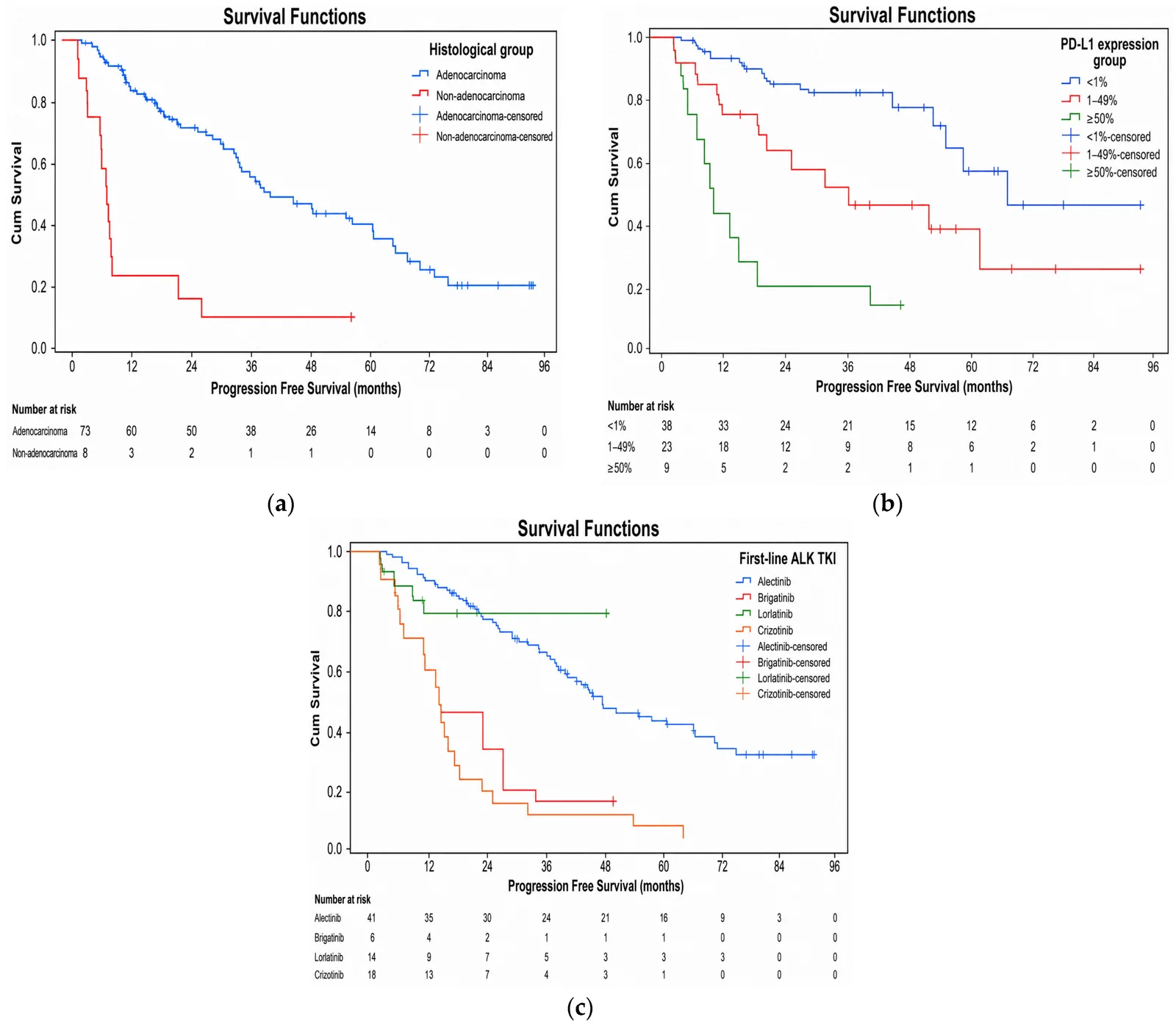
Kaplan–Meier curves for progression free survival according to prognostic factors: (**a**) histological group; (**b**) PD-L1 expression status; and (**c**) first-line ALK TKI selection.

The median PFS in the overall cohort was 32 months (95% CI, 24.13–39.87). Among patients receiving lorlatinib as first-line treatment for metastatic disease, the median PFS was not reached, while the median PFS was 47.5 months (95% CI, 20.51–74.43), 23.0 months (95% CI, 0.00–46.21), and 14.9 months (95% CI, 13.85–15.91) in patients receiving alectinib, brigatinib, and crizotinib, respectively. A statistically significant difference was observed between alectinib and crizotinib (*p* < 0.001) and between alectinib and brigatinib (*p* = 0.045). The 24-month PFS rates were 73.8%, 26.3%, 80.0%, and 42.8% for alectinib, crizotinib, lorlatinib, and brigatinib, respectively. At the time of data cutoff, 56 of 83 patients (67.5%) had experienced a PFS event. The numbers of PFS events were 28 of 42 patients (66.7%) in the alectinib group, 19 of 19 (100%) in the crizotinib group, six of seven (85.7%) in the brigatinib group, and three of 15 (20.0%) in the lorlatinib group. In univariable analyses for PFS, histological subtype (adenocarcinoma vs. non-adenocarcinoma) (*p* = 0.003) and PD-L1 expression status (*p* = 0.040) were statistically significant factors. In multivariable analysis, histological subtype (adenocarcinoma vs. non-adenocarcinoma) (*p* < 0.001) and PD-L1 expression status (*p* = 0.018) remained statistically significant (Table 3). Kaplan–Meier curves of variables with prognostic significance for PFS are presented in Figure 2.

### 3.5. Subsequent Systemic Treatments and Later-Line Lorlatinib

Of the 83 patients, 43 (51.8% of the overall cohort) received second-line systemic therapy after first-line ALK TKI treatment. Among these patients, lorlatinib was the most frequently administered second-line treatment (n = 24, 55.8%), followed by alectinib (n = 16, 37.2%), brigatinib (n = 1, 2.3%), platinum–pemetrexed chemotherapy (n = 1, 2.3%), and carboplatin–paclitaxel chemotherapy (n = 1, 2.3%). Of the 24 patients who received lorlatinib as second-line therapy, 20 (83.3%) had received first-line alectinib and four (16.7%) had received first-line brigatinib. Fourteen patients (16.9% of the overall cohort) received third-line systemic therapy; seven (50.0%) received lorlatinib and seven (50.0%) received platinum–pemetrexed chemotherapy. All seven patients who received lorlatinib as third-line therapy had been treated with crizotinib in the first-line setting followed by alectinib in the second-line setting.

Among patients receiving lorlatinib in subsequent treatment lines, the median PFS-2 was 12.2 months (95% CI, 4.51–19.99) in the second-line setting, whereas the median PFS-3 was 6.1 months (95% CI, 2.33–9.82) in the third-line setting.

### 3.6. Safety

Safety data are summarized in Table 4. Safety analyses included all patients who received each ALK TKI at any line of therapy. Overall, the incidence of any-grade adverse events ranged from 58.6% to 68.4% across the four ALK TKIs. Dose reductions were required in 12.5–36.8% of patients, while treatment discontinuation due to toxicity occurred in only one patient receiving lorlatinib. Cognitive adverse events were observed in six patients (13.0%) treated with lorlatinib, with only one grade 3–4 event. One patient developed grade 4 papilledema, which led to permanent discontinuation of lorlatinib.

## 4. Discussion

In this study, real-world efficacy outcomes, safety profiles, and prognostic factors affecting survival were evaluated in patients with metastatic *ALK*-rearranged NSCLC who received first-line ALK TKI treatment. The median OS of patients receiving alectinib, crizotinib, and brigatinib were 84.8 months, 63.6 months, and 36.8 months, respectively. The median OS was not reached in patients receiving lorlatinib. ECOG PS, histological type (adenocarcinoma vs. non-adenocarcinoma), and PD-L1 status were prominent prognostic factors for OS. The safety profiles of ALK TKIs were consistent with previously reported data, and most adverse events were manageable with dose modifications. Although several real-world studies have previously evaluated outcomes with ALK TKIs, the present study provides complementary evidence by integrating long-term survival outcomes, prognostic factors, treatment sequencing, and safety data within a single real-world cohort. The relatively long follow-up of the overall cohort provides information on long-term outcomes in routine clinical practice, while the inclusion of patients treated with first-, second-, and third-generation ALK TKIs reflects the evolution of ALK-targeted therapy over time. Furthermore, our study provides information on later-line lorlatinib treatment sequences and contributes real-world data from Türkiye, a setting that remains relatively underrepresented in the published literature on *ALK*-rearranged NSCLC.

In the final analysis of the ALEX study published in 2025, the median OS was 81.1 months in patients receiving alectinib and 54.2 months in patients receiving crizotinib [7]. Although the median OS observed in our study was numerically higher than the values reported in the final analysis of the ALEX study, our findings were broadly consistent with the survival outcomes reported in ALEX. In a real-world study conducted by Kamali et al. in Sweden, the median OS was 35 months in the crizotinib group, while the median OS was not reached in patients receiving second-generation ALK TKIs. In this study, 20% of patients had brain metastases at baseline, and 27.3% developed brain metastases during follow-up [14]. Similarly, in a real-world study conducted in Argentina including 106 patients, the second- and third-generation ALK TKI groups did not reach the median OS, but superiority over crizotinib in terms of OS was observed [15]. Kim et al. compared brigatinib and alectinib in a study conducted in Korea. However, the median OS was not reached in either group, and the brigatinib group was reported to have numerically better outcomes in terms of OS [16]. Similarly, in a study conducted in Serbia including 23 patients treated with brigatinib, the median OS was 32 months in patients receiving brigatinib as first-line treatment [17]. In our study, the brigatinib group had a numerically shorter OS than expected. This difference may be associated with the limited number of patients in the brigatinib group, the poorer ECOG performance status, and the higher rate of brain metastases in this group. In the first-line lorlatinib group, median OS was not reached, and the 24-month OS rate was 86.6%; however, given the small sample size and relatively short follow-up, this should be considered a preliminary observation. Importantly, these treatment-specific OS estimates should not be interpreted as evidence of comparative efficacy because of differences in treatment era, follow-up duration, baseline characteristics, and access to subsequent therapies.

In our study, the median PFS was 47.5 months in patients receiving alectinib, 23 months in patients receiving brigatinib, and 14.9 months in patients receiving crizotinib in the metastatic first-line setting. In the PFS analysis of the ALEX study, the median PFS was 34.8 months in the alectinib group and 10.9 months in the crizotinib group [18]. Reale et al. reported a median PFS of 43.1 months for alectinib in their real-world data analysis [19]. For brigatinib, the integrated analysis of the ALTA-1L and J-ALTA studies reported a median PFS of 29.3 [20]. In our study, the PFS duration observed in the alectinib group was numerically favorable compared with previously reported randomized clinical trial and real-world data, whereas a shorter PFS was observed in the brigatinib group. However, treatment-specific outcomes in our cohort should be interpreted cautiously because of the substantial differences in follow-up duration and the non-contemporaneous use of individual ALK TKIs. Although median PFS was not reached in the first-line lorlatinib group and the 24-month PFS rate was 80%, the relatively small sample size and shorter follow-up in this group preclude definitive comparisons with the other ALK TKIs. These findings provide preliminary real-world evidence suggesting durable disease control with first-line lorlatinib but should not be interpreted as evidence of comparative treatment efficacy. In the CROWN study, the median PFS was still not reached at the end of the 7-year follow-up. However, the 24-month PFS rate was 79%, while the 7-year PFS rate was 55% [12]. These preliminary findings are consistent with the favorable disease control observed with lorlatinib in the CROWN study, although longer follow-up is required to assess the durability of this benefit in our real-world cohort.

In patients receiving lorlatinib treatment in the second-line metastatic setting, the median PFS-2 was 12.2 months, while the median PFS-3 was 6.1 months in patients receiving lorlatinib in the third-line setting. In the global phase II prospective study conducted by Solomon et al., the median PFS was reported as 7.3 months in patients receiving lorlatinib in the second and subsequent-line settings and 6.9 months in patients receiving lorlatinib in the third and subsequent-line settings [21]. Kumar et al. reported a median PFS of 16 months for lorlatinib in the second-line setting [22]. In another retrospective study conducted in China, the median PFS in the second-line setting was reported as 12.17 months [23]. In our study, the PFS obtained with lorlatinib in the second-line setting was numerically longer compared with the study by Solomon et al. and was generally consistent with other real-world data studies. However, the PFS durations obtained with lorlatinib in the second and subsequent-line settings did not reach the prolonged PFS benefit achieved with first-line lorlatinib use.

Wang et al. reported that patients with PD-L1 < 1% had significantly better OS and PFS outcomes compared with patients with PD-L1 ≥ 1% in their study [24]. Özdemir et al. reported that PD-L1 ≥ 50% expression was present in 19.75% of 81 patients with ALK rearrangements [25]. In our study, this rate was 12% in the entire cohort and 16.4% among patients with known PD-L1 status. In our study, PD-L1-negative patients had better OS outcomes, while OS progressively worsened with increasing PD-L1 expression levels. Particularly, patients with PD-L1 ≥ 50% had significantly worse outcomes. The same pattern was observed for PFS. These findings suggest that high PD-L1 expression in *ALK*-rearranged NSCLC may be associated with more aggressive tumor characteristics or tumor heterogeneity, independent of immune biology [24,26]. Although *ALK* rearrangements can also be detected in non-adenocarcinoma histologies, studies have reported that these patients have worse outcomes compared with patients with adenocarcinoma histology. This situation has been suggested to be associated with false-positive ALK rearrangement results or higher rates of *EML4-ALK* fusion in this patient group [27,28,29]. However, the exact underlying mechanism remains unclear. In our study, non-adenocarcinoma histologies were associated with significantly worse OS and PFS outcomes compared with adenocarcinoma histology. Although ECOG PS did not show statistically significant prognostic importance for PFS in our study, it was associated with OS outcomes. Conversely, the ALK TKI received in the first-line setting showed a statistically significant difference in terms of PFS but did not demonstrate a significant difference in terms of OS. Although the presence of brain metastases was associated with worse OS in the univariable analysis, this association was no longer statistically significant after multivariable adjustment. This attenuation may reflect the influence of other clinical and prognostic factors included in the model, suggesting that the prognostic effect observed in the univariable analysis was not independent of these covariates. However, the relatively small sample size and limited number of events may also have reduced the statistical power to detect an independent association.

The safety profiles of ALK TKIs observed in our study were generally consistent with the results reported in previous clinical trials and real-world studies. The frequency of cognitive adverse events associated with lorlatinib was similar to previously published [30,31]. Most treatment-related adverse events were grade 1–2 and were manageable with supportive treatments or dose reductions. Treatment was permanently discontinued in only one patient due to grade 4 papilledema. These findings support that currently used ALK TKIs have safe and manageable toxicity profiles in routine clinical practice.

Our study has several limitations. First, its retrospective design inevitably introduced heterogeneity in patient characteristics, follow-up procedures, and treatment management. In addition, the imbalance in the number of patients across treatment groups and the small sample sizes of some subgroups may have reduced statistical power and limited the reliability of between-group comparisons.

The median follow-up duration differed substantially among the first-line treatment groups, reflecting the different periods during which individual ALK TKIs became available in clinical practice. Consequently, the treatment cohorts were not contemporaneous, introducing potential calendar-time bias and differences in access to subsequent therapies. These factors, together with the limited number of patients and events in some treatment groups, particularly the first-line lorlatinib cohort, reduce the comparability and precision of treatment-specific OS and PFS estimates. The relatively low number of deaths in some groups further limits the precision of treatment-specific OS estimates. Therefore, between-treatment comparisons should be interpreted as exploratory rather than as evidence of comparative efficacy. In addition, the limited sample size and marked imbalance across treatment groups restricted the feasibility of applying robust causal inference approaches, such as propensity score-based methods or inverse probability weighting, to further address potential selection bias.

CNS-specific outcomes, including intracranial response and CNS progression, could not be systematically evaluated because these data were not consistently available in the retrospective medical records. Therefore, the present study cannot provide comparative conclusions regarding the intracranial efficacy of individual ALK TKIs.

Subsequent systemic and local therapies may also have influenced OS outcomes and represent an additional source of potential confounding. Data regarding post-progression local treatments, including radiotherapy, stereotactic radiosurgery, stereotactic body radiotherapy, and surgery, were incomplete for a substantial proportion of patients and therefore could not be reliably incorporated into the analysis. Consequently, the potential effects of subsequent systemic and local treatment strategies on treatment-specific OS estimates could not be fully accounted for.

Finally, progression was primarily determined from radiology reports and treating clinician assessments in routine clinical practice. Uniform retrospective RECIST v1.1 reassessment was not feasible because complete radiological imaging was unavailable for some patients, potentially introducing variability in PFS assessment. Larger, multicenter, prospective studies with standardized follow-up and response assessment are needed to validate these findings and improve their generalizability.

## 5. Conclusions

In conclusion, this real-world study provides long-term clinical outcomes and prognostic insights into patients with metastatic *ALK*-rearranged NSCLC treated with ALK TKIs. A numerically longer median OS was observed in the alectinib group than in the crizotinib group (84.8 vs. 63.6 months); however, treatment-specific outcomes should be interpreted cautiously given the differences in treatment era and follow-up duration. ECOG performance status, histological subtype (adenocarcinoma versus non-adenocarcinoma), and PD-L1 expression were independent prognostic factors for OS in patients with *ALK*-rearranged NSCLC. The findings in the first-line lorlatinib subgroup should be considered preliminary because of the small sample size and relatively short follow-up. Nevertheless, the observed PFS findings suggest durable disease control and warrant confirmation in larger real-world cohorts with longer follow-up. More modest PFS outcomes were observed with lorlatinib in later treatment lines.

## Figures and Tables

**Table 1:** Clinicopathological characteristics of 83 patients.

Features	Frequency n (%)				
	All 83 (100)	Alektinib 42 (50.6)	Crizotinib 19 (22.9)	Lorlatinib 15 (18.1)	Brigatinib 7 (8.4)
Gender					
Male	45 (54.2)	24 (57.1)	10 (52.6)	7 (46.7)	4 (57.1)
Female	38 (45.8)	18 (42.9)	9 (47.4)	8 (53.3)	3 (42.9)
ECOG PS ^1^					
0	6 (7.2)	4 (9.5)	1 (5.3)	1 (6.7)	0 (0.0)
1	66 (79.5)	34 (81)	16 (84.2)	12 (80)	4 (57.1)
2	11 (13.3)	4 (9.5)	2 (10.5)	2 (13.3)	3 (42.9)
Smoking status					
Yes	35 (42.2)	20 (47.6)	6 (31.6)	7 (46.7)	2 (28.6)
No	48 (57.8)	22 (52.4)	13 (68.4)	8 (53.3)	5 (71.4)
Histological subtype					
Adenocarcinoma	74 (89.2)	36 (85.7)	19 (100)	13 (86.7)	6 (85.7)
Squamous cell carcinoma	3 (3.6)	2 (4.8)	0 (0.0)	1(6.7)	0 (0.0)
NOS ^2^	4 (4.8)	2 (4.8)	0 (0.0)	1 (6.7)	1 (14.3)
Adenosquamous carcinoma	2 (2.4)	2 (4.8)	0 (0.0)	0 (0.0)	0 (0.0)
Adenocarcinoma subtypes					
Solid predominant	9 (10.8)	3 (7.1)	2 (10.5)	3 (20)	1 (14.3)
Acinar predominant	8 (9.6)	5 (11.9)	1 (5.3)	1 (6.7)	1 (14.3)
Papillary predominant	2 (2.4)	1 (2.4)	1 (5.3)	0 (0.0)	0 (0.0)
Micropapillary predominant	3 (3.6)	2 (4.8)	1 (5.3)	0 (0.0)	0 (0.0)
Signet ring cell	2 (2.4)	0 (0.0)	0 (0.0)	2 (13.3)	0 (0.0)
Unknown	59 (71.1)	31 (73.8)	14 (73.7)	9 (60)	5 (71.4)
Mucin staining					
Positive	32 (38.6)	14 (33.3)	8 (42.1)	9 (60)	1 (14.3)
Negative	30 (36.1)	15 (35.7)	6 (31.6)	4 (26.7)	5 (71.4)
Unknown	21 (25.3)	13 (31.0)	5 (26.3)	2 (13.3)	1 (14.3)
De novo metastasis					
Yes	65 (78.3)	34 (81.0)	12 (63.2)	12 (80)	7 (100)
No	18 (21.7)	8 (19.0)	7 (36.8)	3 (20)	0 (0.0)
Site of metastasis					
Contralateral Lung	15 (18.1)	6 (14.3)	6 (31.6)	2 (13.3)	1 (14.3)
Bone	33 (39.8)	20 (47.6)	5 (26.3)	6 (40.0)	2 (28.6)
Liver	13 (15.7)	8 (19.0)	2 (10.5)	2 (13.3)	1 (14.3)
Brain	23 (27.7)	11 (26.2)	4 (21.1)	5 (33.3)	3 (42.9)
Distant lymph node	33 (39.8)	18 (42.9)	6 (31.6)	6 (40)	3 (42.9)
Pleural metastasis	50 (60.2)	24 (57.1)	13 (68.4)	9 (60)	4 (57.1)
PD-L1 ^3^ groups					
PD-L1 < 1%	39 (47)	23 (54.8)	5 (26.3)	8 (53.3)	3 (42.9)
PD-L1 = 1–49%	24 (28.9)	9 (21.4)	7 (36.8)	5 (33.3)	3 (42.9)
PD-L1 ≥ 50%	10 (12)	6 (14.3)	2 (10.5)	2 (13.3)	0 (0.0)
Unknown	10 (12)	3 (7.1)	5 (26.3)	0 (0.0)	1 (14.3)

^1^ Eastern Cooperative Oncology Group performance status, ^2^ not otherwise specified, ^3^ programmed death-ligand 1.

**Table 2:** Prognostic factors associated with overall survival in univariate and multivariate analyses.

Variable	Category	Median OS (Months)	Univariate HR (95% CI, *p* Value)	Multivariate HR (95% CI, *p* Value)
Gender	Male	70.4	0.76 (0.39–1.50, *p* = 0.434)	
	Female	81.3		
Smoking status	Never smoker	81.3	1.29 (0.66–2.52, *p* = 0.458)	
	Smoker	70.4		
ECOG performance status	ECOG 0–1	81.3	2.69 (1.17–6.20, *p* = 0.020 *)	4.74 (1.84–12.19, *p* = 0.001 *)
	ECOG 2	36.8		
Histological group	Adenocarcinoma	81.3	4.77 (1.88–12.11, *p* = 0.001 *)	5.56 (1.86–16.59, *p* = 0.002 *)
	Non-adenocarcinoma	23.3		
Metastatic status	De novo metastatic	73.9	1.10 (0.51–2.36, *p* = 0.808)	
	Metachronous metastatic	81.3		
Brain metastasis	No	84.8	2.12 (1.06–4.25, *p* = 0.035 *)	1.74 (0.77–3.91, *p* = 0.182)
	Yes	51.3		
Liver metastasis	No	71.2	0.76 (0.27–2.15, *p* = 0.599)	
	Yes	NR		
Pleural metastasis	No	73.9	1.02 (0.52–2.00, *p* = 0.965)	
	Yes	70.4		
Contralateral lung metastasis	No	71.2	1.09 (0.47–2.49, *p* = 0.847)	
	Yes	58.2		
Distant lymph node metastasis	No	67.5	0.67 (0.33–1.35, *p* = 0.266)	
	Yes	NR		
Bone metastasis	No	81.3	1.62 (0.83–3.17, *p* = 0.161)	
	Yes	51.3		
First-line ALK TKI	All patients	73.9	*p* = 0.157	
	Alectinib	84.8	Reference	
	Brigatinib	36.8	3.38 (1.08–10.59, *p* = 0.037 *)	
	Lorlatinib	NR	1.29 (0.28–6.03, *p* = 0.744)	
	Crizotinib	63.6	1.73 (0.83–3.61, *p* = 0.142)	
PD-L1 expression group	All patients	73.9	*p* = 0.004 *	*p* < 0.001 *
	<1%	84.8	Reference	Reference
	1–49%	71.2	2.10 (0.92–4.79, *p* = 0.078 )	2.61 (1.11–6.12, *p* = 0.028 *)
	≥50%	33.4	5.16 (1.94–13.71, *p* = 0.001 *)	8.83 (2.99–26.08, *p* < 0.001 *)

* Represents a significant outcome (*p* < 0.05). ECOG, Eastern Cooperative Oncology Group; HR, hazard ratio; CI, confidence interval; NR, not reached; ALK TKI, anaplastic lymphoma kinase tyrosine kinase inhibitor; PD-L1, programmed death-ligand 1; OS, overall survival.

**Table 3:** Prognostic factors on progression free survival with univariate and multivariate analysis.

Variable	Category	Median PFS (Months)	Univariate HR (95% CI, *p* Value)	Multivariate HR (95% CI, *p* Value)
Gender	Male	27.6	0.67 (0.39–1.15, *p* = 0.143)	
	Female	36.8		
Smoking status	Never smoker	32.7	1.11 (0.65–1.89, *p* = 0.701)	
	Smoker	27.6		
ECOG performance status	ECOG 0–1	32.4	1.84 (0.90–3.78, *p* = 0.095)	
	ECOG 2	14		
Histological group	Adenocarcinoma	34.9	3.25 (1.51–6.99, *p* = 0.003 *)	5.13 (2.09–12.61, *p* < 0.001 *)
	Non-adenocarcinoma	10.9		
Metastatic status	De novo metastatic	32	1.17 (0.64–2.16, *p* = 0.604)	
	Metachronous metastatic	27.7		
Brain metastasis	No	32.7	1.71 (0.96–3.02, *p* = 0.067)	
	Yes	23		
Liver metastasis	No	32.4	1.39 (0.72–2.70, *p* = 0.325)	
	Yes	28.7		
Pleural metastasis	No	29.6	0.94 (0.55–1.60, *p* = 0.819)	
	Yes	34.9		
Contralateral lung metastasis	No	35.9	1.39 (0.71–2.71, *p* = 0.332)	
	Yes	21		
Distant lymph node metastasis	No	28.7	0.86 (0.50–1.48, *p* = 0.586)	
	Yes	34.9		
Bone metastasis	No	32.4	1.50 (0.88–2.55, *p* = 0.134)	
	Yes	28.7		
First-line ALK TKI	All patients	32	*p* < 0.001 *	*p* < 0.001 *
	Alectinib	47.5	Reference	Reference
	Brigatinib	23	2.42 (0.98–5.99, *p* = 0.056)	2.91 (1.03–8.26, *p* = 0.045 *)
	Lorlatinib	NR	0.90 (0.26–3.08, *p* = 0.870)	0.89 (0.25–3.17, *p* = 0.861)
	Crizotinib	14.9	3.59 (1.93–6.67, *p* < 0.001 *)	5.97 (2.80–12.74, *p* < 0.001 *)
PD-L1 expression group	All patients	32	*p* = 0.040 *	*p* = 0.018 *
	<1%	61.6	Reference	Reference
	1–49%	21	1.55 (0.80–2.97, *p* = 0.192)	1.46 (0.75–2.83, *p* = 0.262)
	≥50%	15.3	2.80 (1.25–6.29, *p* = 0.012 *)	3.29 (1.44–7.44, *p* = 0.005 *)

* Represents a significant outcome (*p* < 0.05). ECOG, Eastern Cooperative Oncology Group; HR, hazard ratio; CI, confidence interval; NR, not reached; ALK TKI, anaplastic lymphoma kinase tyrosine kinase inhibitor; PD-L1, programmed death-ligand 1; PFS, progression free survival.

**Table 4:** Summary of treatment-related adverse events.

Safety Outcome	Lorlatinib (n = 46)	Alectinib (n = 58)	Crizotinib (n = 19)	Brigatinib (n = 8)
Adverse events, n (%)				
Any-grade	31 (67.4)	34 (58.6)	13 (68.4)	5 (62.5)
Grade 1	10 (21.7)	18 (31.0)	6 (31.6)	3 (37.5)
Grade 2	16 (34.8)	8 (13.8)	5 (26.3)	2 (25)
Grade 3–4	5 (10.9)	8 (13.8)	2 (10.5)	0 (0.0)
Dose reduction, n (%)	15 (32.6)	16 (27.6)	7 (36.8)	1 (12.5)
Most common adverse event	Hyperlipidemia (39.1%)	Fatigue (22.4%)	Edema (36.8%)	Elevated transaminases (25%)

## Data Availability

The data generated and/or analyzed in this study are included within this article.

## Abbreviations

The following abbreviations are used in this manuscript:

NSCLC	Non-small cell lung cancer
ALK	Anaplastic lymphoma kinase
TKI	Tyrosine kinase inhibitor
OS	Overall survival
PFS	Progression free survival
ECOG PS	Eastern Cooperative Oncology Group performance status
PD-L1	Programmed death-ligand 1
IHC	Immunohistochemistry
FISH	Fluorescence in situ hybridization
NGS	Next-generation sequencing
NOS	Not otherwise specified
CI	Confidence interval
NR	Not reached
HR	Hazard ratio
